# Patient Factors Affecting Physicians’ Decision to Add Perineoplasty to Pelvic Organ Prolapse Surgery: A Quantitative Analysis

**DOI:** 10.3390/jcm15030916

**Published:** 2026-01-23

**Authors:** Esther C. A. M. van Swieten, Yasmina Chaghouaoui, Karlijn J. van Stralen, Jan-Paul W. R. Roovers

**Affiliations:** 1 Department of Obstetrics and Gynecology, Spaarne Gasthuis, 2000 AK Haarlem, The Netherlands; y.chaghouaoui@student.vu.nl (Y.C.); kvanstralen@spaarnegasthuis.nl (K.J.v.S.); 2 Department of Obstetrics and Gynecology, Amsterdam UMC, 1100 DD Amsterdam, The Netherlands; j.p.roovers@amsterdamumc.nl

**Keywords:** perineoplasty, prolapse, surgery, patient factors

## Abstract

**Background/Objectives:** Perineoplasty can be performed as an adjunct to native tissue pelvic organ prolapse (POP) surgery; the optimal indication for perineoplasty is unknown due to limited evidence regarding its benefits and the absence of clear clinical guidelines. This study aims to describe patient-related factors associated with surgeons’ decisions to add perineoplasty to POP surgery and to quantify the frequency of intraoperative changes from preoperative surgical plans. **Methods:** In this multicenter observational cohort study, women ≥ 18 years scheduled for primary native tissue POP surgery between April 2023 and November 2024 were included. Baseline characteristics, pelvic floor anatomy (POP-Q), genital hiatus (GH), perineal body (PB) measurements, and surgeon-reported considerations regarding perineoplasty were collected. Surgical plans (“with”, “without”, or “undecided”) were documented and compared with the actual performed procedure. Logistic and linear regression analyses were used to identify factors associated with perineoplasty. **Results:** Among the 305 enrolled women, 285 underwent surgery, of whom 135 (47%) received perineoplasty. Patients who underwent perineoplasty had a larger GH size (5.2 cm) compared to patients without perineoplasty (4.5 cm). Obesity was associated with an increased rate of perineoplasty compared to normal weight (OR 2.3 95%CI 1.2–4.6). There was a strong exponential association between childbirth and perineoplasty, with a fivefold increase for two children (95%CI 1.3–17.1) and thirtyfold increase for four or more children (95%CI 6.3–142) compared to one child. Nearly all procedures (92%) followed the preoperative plan; surgeons were more likely to omit than add perineoplasty intraoperatively. Surgeons frequently reported GH/PB size and age as key considerations to perform perineoplasty and lack of evidence and fear of dyspareunia as reasons to not perform perineoplasty. **Conclusions:** Surgeons more often perform perineoplasty in patients with factors that have been associated with a higher risk of recurrent prolapse. Prospective comparative studies are required to determine whether perineoplasty reduces recurrent POP after primary surgical repair.

## 1. Introduction

Pelvic organ prolapse (POP) is a common condition that significantly affects women’s quality of life [1,2,3], with an anatomical prevalence estimated at approximately 50% [4] and a symptomatic rate around 11% [5,6]. It incurs considerable healthcare costs [7]. Risk factors for POP include multiple parity, advanced age, and a high body mass index (BMI) [8]. Additionally, conditions such as connective tissue disorders, genetic predisposition, menopause, and prior hysterectomy can increase prolapse risk [9]. The lifetime risk of requiring surgery for POP is estimated at about 12% [6]. A major challenge in pelvic floor surgery is the high rate of recurrent prolapse post-surgery; one in nine women undergoes surgery for POP, but one in five surgical procedures is a repeat operation for recurrent POP [10].

To restore pelvic floor function, vaginal support needs to be reinforced. The vagina is, according to DeLancey, supported at three levels [11].

- Level I: The upper suspensory ligaments (apical support of the vagina).
- Level II: The pubocervical and rectovaginal fasciae (lateral support of the vagina).
- Level III: The pelvic floor muscles of the levator ani (support of the vagina to the perineal body).

Loss of level I support leads to prolapse of the uterus or vaginal vault (in the case of a previous hysterectomy); level II defects lead to recto-, entero-, and cystoceles; and insufficient level III support leads to a wide genital hiatus (GH), which correlates with a higher risk of level I defects [12] and a higher risk of recurrent prolapse after surgery [13,14].

Worldwide clinical practice has mainly focused on level I and level II repair, about which there is an abundance of research and solid evidence on which intervention should be performed for which indication. In recent years, more research has been published on level III and perineoplasty [15], indicating that performing perineoplasty leads to a smaller GH and better quality of life [16] and that a reduced GH after perineoplasty persists postoperatively [17].

However, there still is a paucity of evidence on the exact added value of surgery to address level III support in the case of a wide GH, which results in significant practice variation [18]. This reflects a significant disparity in clinical practices due to the absence of standardized protocols guiding when to incorporate perineoplasty into surgical plans [19]. Therefore, this study aims to describe [1] the patient-specific factors that influence surgeons’ decisions to add perineoplasty during pelvic organ prolapse surgery and [2] the frequency of changes in surgical plans throughout the intervention compared to preoperative discussions.

## 2. Materials/Patients and Methods

### 2.1. Study Design

We conducted a multicentre, prospective, observational cohort study in which we enrolled women aged 18 years and older who were scheduled for primary native tissue POP surgery, with or without perineoplasty. The study represents a baseline analysis within a larger investigation aimed at determining the success of adding perineoplasty to POP surgery.

Patients were recruited from 10 teaching hospital in the Netherlands from April 2023 to November 2024. Before starting the study, we organized a consensus meeting to determine the optimal surgical technique for perineoplasty. An instructional video was created for training purposes so that all surgeons would perform perineoplasty in exactly the same way. During the same meeting, we discussed the threshold for GH. Several studies state that a GH of <4 cm is considered not to be enlarged [20,21]; thus, there is no indication for perineoplasty and a consensus was reached that a GH above 7 cm is always an indication of enlargement. The study protocol was approved by the Medical Ethics Review Committee (METC) under file number NL2022.0704—NL82381.018.22 and registered at ClinicalTrials.gov under number NCT05713422. All participants provided written informed consent prior to inclusion.

### 2.2. Study Population

Eligible adult participants included women indicated for primary native tissue POP surgery with a GH of ≥4 cm and ≤7 cm. Patients who were (intending to become) pregnant, had a previous POP surgery (native tissue as well as mesh-augmented), and who were unable to understand Dutch were excluded since all validated questionnaires were in Dutch. Previous incontinence surgery was not an exclusion criterion.

### 2.3. At Baseline

Surgeons documented participant characteristics including age, height, weight, body mass index (BMI), smoking status, parity, mode of delivery, history of third- or fourth-degree perineal rupture, previous non-surgical treatment, and previous hysterectomy. Pelvic floor anatomy was assessed using the Pelvic Organ Prolapse Quantification (POP-Q) staging system, and 2D ultrasound imaging was used to measure the anteroposterior distances from the puborectalis muscle to the symphysis. This ultrasound was performed under Valsalva, with the ultrasound probe placed mid-sagittal against the perineum visualizing the symphysis, urethra, bladder neck, vagina, cervix, anal canal, and levator ani. Baseline records also included patient reports of pain and dyspareunia.

Surgical plans were established during the initial visit through shared decision-making, where the surgeons documented whether surgery was planned “with perineoplasty” or “without perineoplasty” or whether the decision remained “undecided”. Surgeons were prompted to record influences on their decisions regarding perineoplasty from a predefined list of considerations, which included age, GH size, pelvic anatomy, concerns about dyspareunia, sexual activity, pain, the absence of evidence supporting the procedure, or other rationales.

Postoperatively, data regarding whether perineoplasty was actually performed (yes or no) were recorded, alongside surgeons’ considerations leading to the final decision. Surgeons could select multiple considerations.

### 2.4. Statistical Analysis

Demographic and clinical characteristics were analyzed to provide an overview of the study population at baseline. Comparisons were made between groups categorized by planned surgical approach, namely the group with planned perineoplasty and the group without planned perineoplasty using logistic regression analyses. Multivariate analyses were performed using generalized linear models with centre as a level, and we adjusted all analyses for BMI group and parity.

All statistical analyses were conducted using SPSS (version 26). A *p*-value < 0.05 was considered statistically significant.

## 3. Results

**Figure 1:**
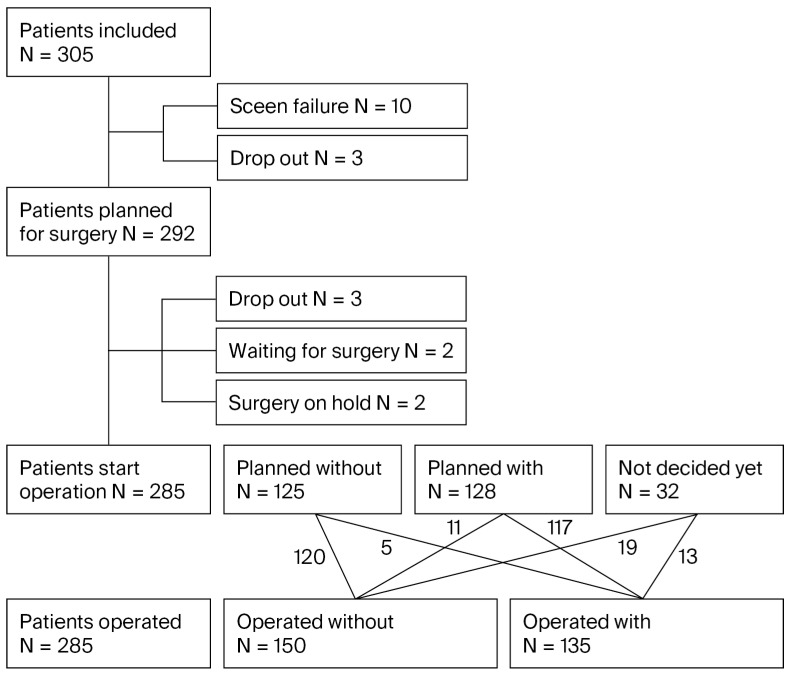
Flow chart of study population from inclusion to actual performed surgery.

A total of 305 women were included, of whom 292 were planned for surgery and 285 received surgery. Of these women, 135 received perineoplasty, while 150 did not receive perineoplasty (Figure 1).

### 3.1. Patient Factors Associated with Performing Perineoplasty (Table 1)

The mean age among the women who had received perineoplasty was not significantly different from those who did not receive perineoplasty. Between the groups, there were no differences in the mode of delivery (mainly spontaneous vaginal without forceps or vacuum). Nearly 80% in both groups had previous non-surgical treatment, mainly pessaries, while a quarter of women in both groups underwent physiotherapy. Pelvic pain and pre-existent dyspareunia were not different between the groups.

**Table 1:** Patient factors related to actual performed surgery. The multivariate analyses were adjusted for centre, BMI group, and parity.

	Demographics		Risk		
	Performed Surgery				
Patient Factor	(+) Perineoplasty	(−) Perineoplasty	*p*-Value	OR (95% CI) or Difference (95% CI)	OR (95% CI) Adjusted
Total (n)	135	150			
Age, median (P5-PG5)	61.0	63.0	0.24	−1.4 y	0.98
	(39.6–78.2)	(46.5–76.6)		(−3.6–0.95)	(0.96–1.01) per year
BMI, n (%)					
18.5–24.9	47 (35)	71 (47)		1 (ref)	1 (ref)
25.0–29.9	55 (41)	59 (39)	0.20	1.4 (0.8–2.4)	1.5 (0.9–2.4)
≥30.0	31 (23)	20 (13)	0.01	2.3 (1.2–4.6)	2.0 (0.8–4.7)
Smoking, n (%)					
No	102 (84)	100 (74)		1 (ref)	1 (ref)
Yes	3 (3)	4 (3)	0.47	0.6 (0.1–2.7)	0.99 (0.2–5.3)
Former smoker Unknown, missing	17 (14) *13*	32 (24) *14*	0.04	0.5 (0.3–1.0)	0.5 (0.3–0.8)
Parity, n (%)					
0	0	2 (1)	NA	NA	NA
1	3 (2)	18 (12)	ref	1 (ref)	1 (ref)
2	70 (52)	87 (59)	0.01	4.8 (1.3–17.1)	4.7 (1.7–12.8)
3	37 (27)	35 (24)	0.01	6.3 (1.7–23.4)	6.2 (2.3–17.1)
≥4	25 (19)	5 (3)	<0.01	30.0 (6.3–142)	28.5 (6.8–120)
Mode of delivery, n (%)					
Spontaneous vaginal	117 (84)	123 (88)		1 (ref)	1 (ref)
without forceps or vacuum					
Cesarean only no vaginal	0 (0)	1(1)	-	NA	NA
Vaginal forceps no vacuum	4 (3)	4 (3)	0.95	1.1 (0.3–4.3)	1.3 (0.3–6.4)
Vaginal vacuum no forceps	14 (10)	11 (8)	0.65	0.83 (0.4–1.9)	1.0 (0.4–2.5)
Other, none of above	5 (3)	0 (0)	-	NA	NA
Third/fourth degree rupture (+), n (%)	8 (6)	8 (6)	0.88	1.1 (0.4–3.0)	0.8 (0.3–2.1)
Previous non-surgical treatment, n (%)					
No	28 (21)	35 (23)		1 ref	1 (ref)
Pelvic floor physiotherapy	19 (14)	21 (14)	0.76	1.1 (0.5–2.5)	1.1 (0.5–2.6)
Pessary	56 (42)	60 (40)	0.62	1.2 (0.6–2.2)	1.3 (0.6–2.7)
Physiotherapy + pessary	31 (23)	31 (21)	0.53	1.3 (0.6–2.5)	1.2 (0.6–2.1)
Unknown	1 (1)	3 (2)	0.41	0.4 (0.0–4.2)	0.1 (0.0–0.3)
Previous hysterectomy (+), n (%)	11 (8)	6 (4)	0.375	2.1 (0.8–5.8)	2.0 (0.7–6.2)
Genital hiatus (GH), mean ± SD	5.2 ± 0.83	4.5 ± 0.61	<0.01	0.7 cm difference (0.5–0.9)	OR 3.67 (1.9–8.5) per cm
Perineal body (PB), median, mean ± SD	2.9 ± 0.73	3.0 ± 0.72	0.04	−0.2 cm difference (−0.4–0.0)	OR 0.71 (0.46–1.1) per cm
Cystocele (POP-Q), n (%)					
0	4 (3)	5 (3)		1 (ref)	1 (ref)
1	9 (7)	14 (9)	0.78	0.8 (0.2–3.8)	0.8 (0.2–3.1)
2	62 (46)	65 (44)	0.80	1.2 (0.3–4.6)	1.3 (0.6–2.9)
3–4	59 (44)	65 (44)	0.86	1.1 (0.3–4.4)	1.1 (0.4–2.9)
Descensus uteri/vaginal vault prolapse (POP-Q), n (%)					
0	52 (39)	70 (47)		1 (ref)	1 (ref)
1	20 (15)	18 (12)	0.28	1.5 (0.7–3.1)	1.6 (0.7–3.8)
2	39 (29)	47 (32)	0.70	1.1 (0.6–1.9)	1.3 (0.7–2.6)
3–4	24 (18)	14 (9)	0.03	2.3 (1.1–4.9)	3.3 (1.3–7.9)
Rectocele (POP-Q), n (%)					
0	13 (10)	26 (17)		1 (ref)	1 (ref)
1	35 (26)	57 (38)	0.61	1.2 (0.6–2.7)	1.4 (0.7–2.8)
2	73 (54)	53 (36)	0.01	3.3 (1.3–5.9)	3.6 (1.7–7.7)
3–4	14 (10)	13 (9)	0.14	2.2 (0.8–5.9)	2.8 (1.2–6.5)
Pelvic pain (+), n (%)	16 (12)	19 (13)	0.82	0.9 (0.5–1.9)	1.1 (0.6–1.8)
Dyspareunia (+), n (%)	17 (14)	26 (19)	0.26	0.7 (0.4–1.3)	0.60 (0.3–1.1)

However, women who received perineoplasty were significantly more often obese (OR 2.3 95%CI 1.2–4.6) and were less often former smokers (OR 0.5 95%CI 0.3–1.0). Parity was strongly associated with perineoplasty, with women with two children having a 5-fold increase (95%CI 1.3–17.1), those with three children having a 6-fold increase (95%CI 1.7–23.4), and those with four or more having a 30-fold increase (95%CI 6.3–142) in the odds of perineoplasty compared to women with only one child. When we adjust this for BMI, the association is still significant.

GH was significantly larger among patients indicated for a perineoplasty compared to those without, while there was only a slight difference in size of the PB, which we consider as clinically irrelevant. Adjusting for BMI and parity did not change this relation.

Advanced stages of descensus uteri/vaginal vault prolapse (stages 3–4) and rectocele stage 2 were associated with an increased likelihood of performing perineoplasty, while cystocele was not an indication to perform the procedure.

### 3.2. Changes in Intended Surgical Procedure (Figure 1)

At the baseline visit, the surgical plan was established. In 128 patients (45%), the surgeon intended to perform perineoplasty, and in 125 patients (44%), no perineoplasty was planned. In 32 patients (11%), a decision had not been reached yet. Nearly all patients followed the original plan. Of the group planned with perineoplasty, 11 patients (8%) finally underwent surgery without perineoplasty instead. Conversely, in most women planned for surgery without perineoplasty, the initial plan was followed, with only five patients (3%) receiving perineoplasty during surgery. Among the women in the “not decided” group at baseline, 58% ultimately had surgery without perineoplasty, while 42% underwent surgery with perineoplasty.

### 3.3. Rationale for the Performance of Perineoplasty (Figure 2)

Both during the initial visit as well as after surgery, the surgeons were asked for their rationale for performing (or not performing) perineoplasty. Among those patients planned for perineoplasty, the size of the GH or PB (85%), sexual activity (41%), and age (36%) were most often reported. Among those not planned for perineoplasty, results were very similar, with the size of the GH/PB (73%) and sexual activity (55%) being deciding factors. Additionally, concerns about the development of dyspareunia (54%), lack of evidence (30%), and pain complaints (30%) were also frequently reported.

**Figure 2:**
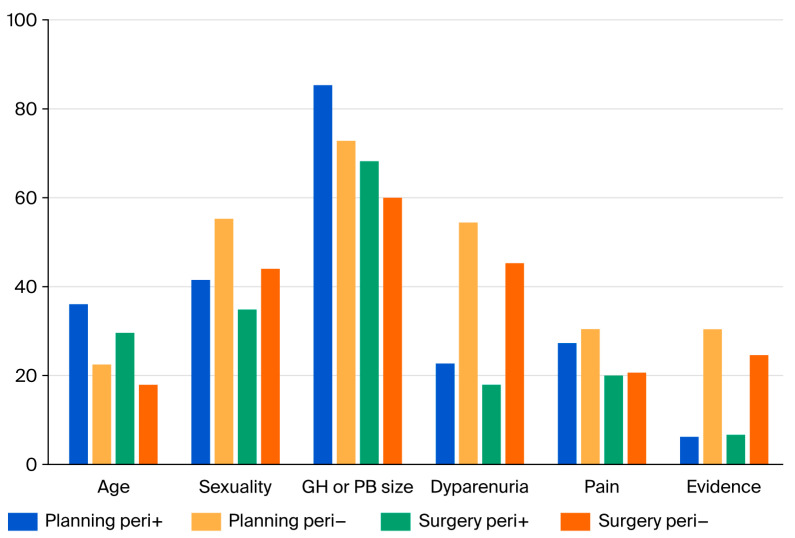
Percentage of patients in whom the surgeon reported an item as important in the decision to (not) perform perineoplasty (peri +/peri −) at the moment of surgical planning (planning) or at the moment of actual surgery (surgery); surgeons were free to select multiple considerations.

## 4. Discussion

In this multicentre, prospective, observational study, we identified the factors surgeons consider in their decision to perform or not perform perineoplasty during vaginal prolapse surgery. Perineoplasty was more often performed among patients with a larger GH size, obesity, higher parity, presence of a rectocele, and more advanced POP stage of the apical compartment.

To our knowledge, no previous studies have reported which factors influence the indication to perform perineoplasty, but the factors we have identified are all known to increase the risk of prolapse recurrence. Thus, our study shows that surgeons may consider perineoplasty as a strategy to reduce the risk of recurrent prolapse after surgery.

Vaughan et al. [20] demonstrated that a larger preoperative GH that is not corrected during surgery is associated with higher surgical failure rates. Similarly, advanced stages of apical compartment prolapse are recognized as risk factors for recurrent prolapse [13], as is an increased BMI [8]. Furthermore, multiparity is a well-established risk factor for the development of POP [13]. Surgeons apparently translate this increased risk into an indication for perineoplasty and consider this procedure to be a method that reduces the risk of recurrence.

This is supported by recent studies that suggest a positive effect of performing perineoplasty in the case of an enlarged GH. Hill et al. [21] recently described that early postoperative GH < 4 cm was associated with superior long-term objective success, without increasing dyspareunia. These data support correcting GH to <4 cm during prolapse repair, as do two other studies [22,23], which performed a retrospective study in which they divided their patients into two groups, one with patients who had a persistently wide GH and one with improved or stable normal GH after surgery; the authors found that there was an increase in anatomic failure after surgery in the persistently wide group.

Although patient age was frequently cited by surgeons as a reason to plan and perform perineoplasty, strikingly, there were no significant age differences between patients who underwent perineoplasty and those who did not. A recent study showed that younger age is a well-established risk factor for POP recurrence [13] and, therefore, one might expect that perineoplasty would be more liberally performed in younger populations. However, we hypothesize that the decision to perform perineoplasty in younger patients may be explained by a more cautious approach in younger patients because of the potentially higher risk of dyspareunia or sexual problems in this group [23].

In addition, surgeons reported fear of developing dyspareunia as an important argument to not perform perineoplasty, possibly because of concerns about deterioration in sexual function after perineoplasty. Surprisingly, no difference in the rate of pre-existing dyspareunia was observed between the group with perineoplasty and the group without perineoplasty.

In the literature, we found inconsistent results regarding the relationship between perineoplasty and dyspareunia. Some studies report an increased risk of de novo dyspareunia [24], while others found an improvement in sexual function after perineoplasty in the case of a wide genital hiatus [25,26] or at least no deterioration when correcting an enlarged GH by performing perineoplasty [21]. Therefore, further research will have to determine the impact of perineoplasty on sexual function and whether this impact is negative or positive.

Interestingly, former smoking status was significantly associated with a lower likelihood of undergoing perineoplasty. A higher proportion of former smokers were initially planned for surgery without perineoplasty compared to non-smokers, and this trend remained during the intraoperative phase. There was no difference in effect estimate between the current and former smokers; however, the number of current smokers was very small and, therefore, the effect was not statistically significant. Smoking has been identified as a protective factor for developing prolapse [13], which might explain why surgeons are more reluctant to perform perineoplasty; however, since our population already had a prolapse, this argument would not be correct. Moreover, smoking has been linked to impaired wound healing and an increased postoperative complication rate [27], suggesting a possible reason for the reluctance to perform perineoplasty on smokers.

Women with a history of hysterectomy had a twofold chance of having perineoplasty planned preoperatively, although this association was not statistically significant. The small number of women with a prior hysterectomy limits the ability to draw firm conclusions with respect to the prognostic value of a previous hysterectomy; this might be an area for future research.

In terms of variations between the surgical plan and performance, most surgical procedures remained consistent with the preoperative plan. There was a slight tendency for intraoperative decisions to exclude perineoplasty rather than add it. Nearly all patients initially planned without perineoplasty underwent the procedure as planned, whereas some patients originally scheduled for perineoplasty ultimately did not undergo the procedure. Thus, we can conclude that surgeons almost never adjust the plan made preoperatively, which may be explained by the fact that surgery does not generate better insights into anatomy as well as a pelvic examination does. Nevertheless, there is a potential risk of confirmation bias, whereby surgeons may (subconsciously) interpret intraoperative findings in line with preoperative expectations: future studies could mitigate this through objective intraoperative criteria, and/or prospective study protocols.

This study’s strengths include its prospective multicentre design, which enhances generalizability, and the use of standardized pelvic floor measurements (POP-Q, genital hiatus, and perineal body). The documentation of both preoperative surgical plans and intraoperative decisions provides valuable insight into real-world surgical decision-making. Additionally, the relatively large cohort strengthens the robustness of the analyses and makes it possible to systematically document a large set of data. Several limitations should be considered. The observational design does not allow for causal inference, and (individual) surgeon-related factors such as experience, case volume, and institutional preferences were not formally assessed, despite the adjustment for centre. Moreover, the relatively small numbers of women reporting dyspareunia may have limited the ability to detect associations with sexual symptoms. Postoperative outcomes were not included in this baseline analysis and are needed to assess the clinical impact of perineoplasty. We will conduct a follow-up of at least 24 months to draw conclusions about the clinical impact of the procedure.

We conclude that surgeons tend to perform perineoplasty in patients that are at higher risk of recurrent prolapse. Retrospective studies indicate that not correcting an enlarged GH during prolapse surgery may lead to an increased risk of surgical failure. Therefore, prospective comparative studies need to show that this indication to perform perineoplasty results in better objective and subjective surgical outcomes. Such studies need to balance the additional morbidity related to perineoplasty against the potential benefits, and ultimately, this will facilitate the shared decision process with respect to surgical planning. The results of our study help pelvic floor surgeons to be more aware of what they intend to achieve in POP surgery when deciding whether to perform perineoplasty.

## Data Availability

The original contributions presented in this study are included in the article. Further inquiries can be directed to the corresponding author.
